# Intrinsic Relations Between Transmission and Reflection in Metamaterials

**DOI:** 10.3390/mi17040493

**Published:** 2026-04-17

**Authors:** Boli Xu, Renbin Zhong

**Affiliations:** Terahertz Research Center, School of Electronic Science and Engineering, University of Electronic Science and Technology of China, Chengdu 610054, China; 202211022430@std.uestc.edu.cn

**Keywords:** metamaterials, artificial structure, electromagnetic waves, transmission-reflection coupling, amplitude-phase coupling

## Abstract

Metamaterials possess high freedom on structural design, yet their ability to modulate electromagnetic waves is subject to intrinsic constraints that are independent of specific meta-atom geometries. The constraints are revealed by analyzing the statistical amplitudes and phases of transmission and reflection wave in some representative metamaterials. Based on scattering theory, a reconstructed and more general description of the electromagnetic modulation process in metamaterials is established. Two explicit and geometry-independent corollaries concerning the coupling between transmission and reflection waves are further obtained and verified. The results provide a new perspective on the fundamental modulation mechanism of metamaterials on electromagnetic waves.

## 1. Introduction

In recent years, metamaterials—artificially engineered materials composed of designed meta-atoms—have become widely recognized [1]. Among them, electromagnetic metamaterials, which are employed to manipulate various properties of electromagnetic waves, occupy a prominent position [2]. Owing to their high design freedom and flexible electromagnetic control, electromagnetic metamaterials have been applied across diverse fields. Representative examples include absorbers used as stealth layers in antenna and radar applications, such as the absorber proposed by Yong Zhi Cheng [3] and by Long Yang [4]; optical imaging devices, including the invisibility cloak demonstrated by D. Schurig et al. [5] and the perfect lens with focusing functionality proposed by Wei-Ting Chen et al. [6]; and information-related applications, such as information-carrying metamaterials proposed by Tie Jun Cui [7], intelligent programmable metamaterials developed by Qian Ma et al. [8], and multilayer metasurfaces functioning as physical neural networks introduced by Che Liu et al. [9].

Across these seemingly diverse metamaterial devices, several non-negligible yet peculiar characteristics are implicitly manifested. For instance, in the frequency-selective surface (FSS) designed by Yunus E. et al. that enhances the performance of broadband reconfigurable antenna apertures [10], all reflection peaks are found to share the same phase value of π. In particular, for reflection spectra exhibiting three distinct resonance peaks, the phase of each peak remains π, hinting that reflection maxima in such metamaterials are associated with a fixed phase value.

This behavior is not an isolated case. In the dielectric Huygens’ surfaces designed by Manuel Decker et al., the transmission peak also occurs at a specific phase of π [11]. Similarly, for the rod antenna metasurface designed by Nanfang Yu et al., the transmission peak corresponds to a phase of π/2 [12]. Notably, in all these cases, the peak phase appears near the midpoint of the overall phase distribution. Furthermore, in the birefringent reflect array metasurface proposed by Mohsen Farmahini-Farahani et al., it is observed that when structural parameters are varied, the transmission peak shifts in frequency and the zero-phase point shifts accordingly [13]. These observations collectively suggest that transmission/reflection amplitudes and their corresponding phases are constrained by an underlying relationship.

Consistent with this intrinsic constraint, the transmission phase of single-layer metamaterials is typically confined within the range of −90° to 90°, and phases beyond this range are observed only in sufficiently thick structures where multi-interface effects become significant [14]. Correspondingly, reported phase-gradient metamaterials are almost exclusively realized using multilayer architectures [15].

All these features indicate that a single-layer metamaterial does not possess unlimited capability in modulating electromagnetic waves. Instead, the achievable amplitudes and phases of transmitted and reflected waves must satisfy certain intrinsic rules that cannot be broken by structural variations alone, unless a single-layer structure is extended to a multilayer configuration with multiple coupled resonances.

Existing descriptions based on S-parameters, transmission-line models, and transfer-matrix methods [16] mainly impose general constraints such as energy conservation and reciprocity or relate transmission and reflection indirectly through intermediate parameters (e.g., effective impedance), and they are typically associated with multilayer coupling effects. However, an explicit and direct constraint on the amplitudes and phases of transmitted and reflected waves at the single-layer level has not been clearly established. These rules have not yet been explicitly identified nor recognized as possessing independent physical significance.

In this study, these intrinsic rules are explicitly identified and formulated as direct coupling relations between the amplitudes and phases of transmitted and reflected waves at the single-layer level.

To address this gap, in this study, we systematically summarize the value distributions of reflection and transmission waves in metamaterials, demonstrating that the amplitudes and phases of incident, transmitted, and reflected waves are not arbitrary. While these distributions appear distinctive, they remain fully consistent with the scattering theory, with periodic structures making these effects more evident for analysis. The interaction between metamaterials and electromagnetic waves is systematically analyzed using Green’s function, allowing us to provide a reconstructed and more general description of the metamaterial-based electromagnetic modulation, which can deepen the understanding of metamaterial behavior and enrich the fundamental theory of metamaterials.

Moreover, two explicit corollaries are derived: an amplitude-phase relation for reflected and transmitted waves and a transmission–reflection relation, with their physical significance, observable phenomena, and potential applications analyzed and the corollaries validated using several representative metamaterials.

This study provides a clearer physical understanding of wave modulation in micro-/nano-structured media, which may offer theoretical guidance for the design of micro/nano electromagnetic devices such as metasurfaces, nano-resonators, and integrated photonic components.

## 2. Relation Analysis of the Amplitudes and Phases of Reflection and Transmission Coefficients

**Figure 1:**
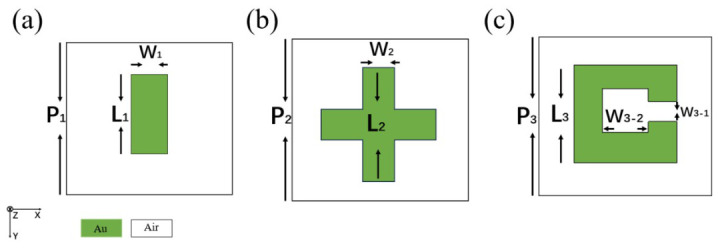
Unit structures of metamaterial. (**a**) Strip-shaped metallic meta-atom with $P_{1}=$ 80 μm, $L_{1}=50\;\mu$m, and $W_{1}=8\;\mu$m. (**b**) Cross-shaped meta-atom with $P_{2}=80\;\mu$m, $L_{2}=50\;\mu$m, and $W_{2}=8\;\mu$m. (**c**) Split ring meta-atom with $P_{3}=40\;\mu$m, $L_{3}=15\;\mu$m, $W_{3-1}$=2.4μm, and $W_{3-2}=10.2\;\mu$m.

In this section, three representative metamaterial structures are selected for an analysis of the amplitudes and phases of their reflection and transmission coefficients. The meta-atom geometries are shown in Figure 1. The green regions denote the metallic components (Au), while the white regions represent air.

**Figure 2:**
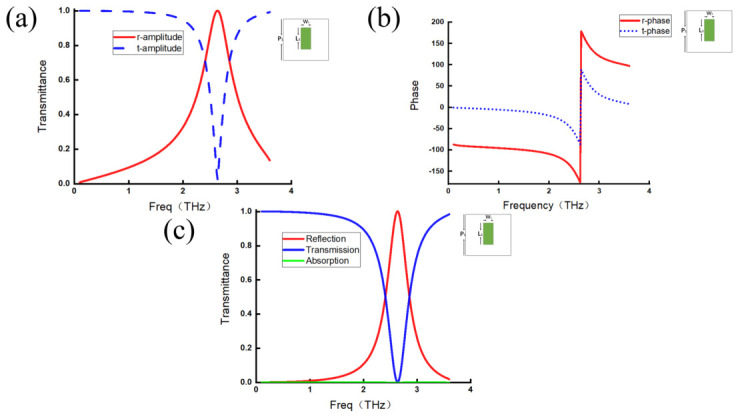
Strip-shaped metallic metamaterials. (**a**) Reflection coefficient amplitude curve (red line) and transmission coefficient amplitude curve (blue line). (**b**) Reflection coefficient phase curve (red line) and transmission phase coefficient curve (blue line). (**c**) Reflection efficiency curve (red line), transmission efficiency curve (blue line), and absorption efficiency curve (green line).

For the strip-shaped metallic meta-atom metamaterial shown in Figure 1a, the spectra of the transmission and reflection coefficients, their corresponding phase responses, and the transmission and reflection efficiencies can be obtained by a simulation, as presented in Figure 2. The red curves denote reflection, while the blue curves denote transmission. Figure 2a,c show the transmission/reflection coefficients and transmission/reflection efficiencies; it can be observed that the positions of the transmission minima coincide with those of the reflection maxima, which is an evident consequence of energy conservation. As for the phase responses shown in Figure 2b, the transmission phase is confined within a range from −90° to +90°, whereas the reflection phase is distributed within intervals of −180° to −90° and 90° to 180°. They have a similar variation trend along frequencies, and the absolute phase difference between them remains constant, revealing nontrivial behavior.

In addition, abrupt phase changes (when the phase is within the interval from $-180^{\circ }$ to $180^{\circ }$) are observed at the transmission minima/reflection maxima. A specific relationship between phases and the corresponding amplitudes can be observed. Following a cosine relationship, the transmission phase reaches $\pm 90^{\circ }$ at zero amplitude, while the reflection phase reaches $\pm 180^{\circ }$ at unit amplitude.

**Figure 3:**
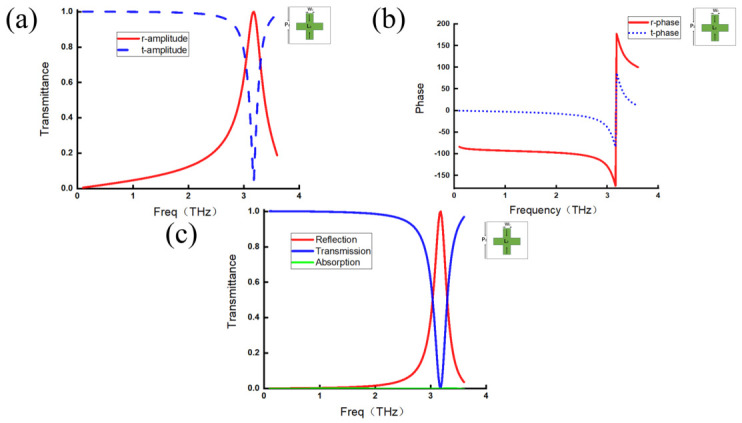
Cross-shaped metamaterials. (**a**) Reflection coefficient amplitude curve (red line) and transmission coefficient amplitude curve (blue line). (**b**) Reflection coefficient phase curve (red line) and transmission phase coefficient curve (blue line). (**c**) Reflection efficiency curve (red line), transmission efficiency curve (blue line), and absorption efficiency curve (green line).

**Figure 4:**
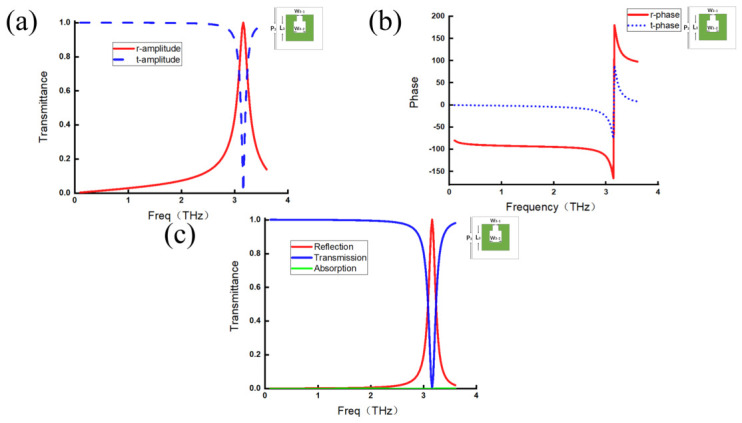
Split ring metamaterials. (**a**) Reflection coefficient amplitude curve (red line) and transmission coefficient amplitude curve (blue line). (**b**) Reflection coefficient phase curve (red line) and transmission phase coefficient curve (blue line). (**c**) Reflection efficiency curve (red line), transmission efficiency curve (blue line), and absorption efficiency curve (green line).

The same analysis is performed for the other two metamaterial structures, with the results shown in Figure 3 and Figure 4. The amplitudes and phases of the transmitted and reflected waves remain consistent with the previously identified characteristics, indicating that these features are universal.

**Figure 5:**
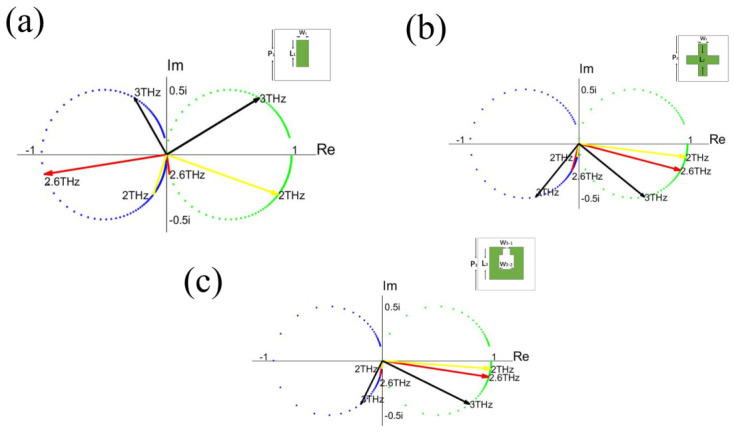
The complex plane distribution diagram of the transmission and reflection coefficients. (**a**) Strip-shaped metallic metamaterials. (**b**) Cross-shaped metamaterial. (**c**) Split ring metamaterials.

To illustrate the distributions and relationships of the amplitudes and phases of the transmission/reflection coefficients more clearly, the coefficients within the relevant frequency band are described in the complex plane, as shown in Figure 5. The blue dots correspond to reflection coefficients, while green dots correspond to transmission coefficients for a different frequency. All the above-mentioned three metamaterial structures exhibit identical features: the reflection coefficients form a circular distribution on the left side of the imaginary axis, while the transmission coefficients form a circular distribution on the right side, further confirming the cosine-type relationship between the amplitudes and phases of the transmission/reflection coefficients intuitively.

Furthermore, three representative frequency points are marked in Figure 5: 2 THz (yellow arrows), 2.6 THz (red arrows), and 3 THz (black arrows). A notable property can be observed: at a certain frequency, the arrow of the reflection coefficient in the blue circle is always perpendicular to that of the transmission coefficient in the green circle, indicating their phase difference—maintaining a fix value of 90°. Apparently, these phenomena are not accidental but rather an inevitable consequence of electromagnetic wave modulated by metamaterials.

From a physical perspective, both transmitted and reflected electromagnetic waves can be regarded as originating from resonant dipoles excited by the incident wave in metamaterials. It can be defined as material scattering phenomena and be described by Green’s function formulations in electromagnetism.

The scattering theory already contains nontrivial first-order field correlations beyond energy-conservation constraints. A representative example is the extinction theorem [17,18,19], which shows that the internal field arises from dipole radiation that simultaneously cancels the incident wave in free space and generates a propagating field inside the medium. This directly implies intrinsic first-order relations among incident, reflected, and transmitted waves.

The correlations identified between the transmission and reflection coefficients of metamaterials therefore originate from fundamental scattering physics encoded in the Green’s function framework. While such effects are typically negligible in conventional materials, metamaterials amplify them to an observable level, making their physical significance explicit and requiring a systematic theoretical formulation.

## 3. Derivation of Field Relations and Physical Picture of Modulation

### 3.1. Relation Derivation Based on Green’s Function

Fundamental equations governing the observed relations in metamaterials can be derived from the Green’s function. The basic solution expression for the Green’s function is given in [19]:

(1) $$\begin{array}{c} U\left(r\right)=U^{\left(i\right)}\left(r\right)+\int U\left(r^{\prime }\right)F\left(r^{\prime }\right)G\left(r-r^{\prime }\right)d^{3}r^{\prime } \end{array}$$

where *U* denotes a specific component of the total electric field, $U^{\left(i\right)}$ denotes a specific component of the incident electric field, r represents the observation position in the observer’s reference frame, $r^{\prime }$ denotes the source position, *F* is the scattering potential of the material, and *G* is the Green’s function.

Expanding Equation (1) with the Liouville–Neumann series and retaining its integral representation [19], one obtains:

(2) $$\begin{array}{cc} U\left(r\right) & =U^{\left(i\right)}\left(r\right)+\int U^{\left(i\right)}\left(r^{\prime }\right)F\left(r^{\prime }\right)G\left(r-r^{\prime }\right)d^{3}r^{\prime }\\ \; & +\int \int U^{\left(i\right)}\left(r^{\prime }\right)F\left(r^{\prime }\right)G\left(r^{\prime }-r^{\prime \prime }\right)F\left(r^{\prime \prime }\right)G\left(r-r^{\prime \prime }\right)d^{3}r^{\prime }{d^{3}r}^{\prime \prime }+\cdots \end{array}$$

This expression explicitly decomposes multiple scattering processes into successive orders: the first term represents zeroth-order scattering, the second term is first-order scattering, the third term is second-order scattering, and so forth.

When Equation (2) is applied to a single-layer metamaterial, each scattering order can be attributed to contributions of individual meta-atoms. Treating each meta-atom as a scattering unit and assuming a lattice period p, Equation (2) can be rewritten as:

(3) $$\begin{array}{cc} U\left(r\right)\; & =U^{\left(i\right)}\left(r\right)+\sum U^{\left(i\right)}\left(np\right)F_{meta}\left(np\right)G_{meta}\left(r,np\right)\\ \; & +\sum \sum U^{\left(i\right)}\left(np\right)F_{meta}\left(np\right)G_{meta}\left(np,mp\right)F_{meta}\left(mp\right)G_{meta}\left(r,mp\right)+\cdots \end{array}$$

In Equation (3), the second term represents the first-order response of the meta-atoms, the third term represents the second-order response, and so forth.

By explicitly isolating the radiation emitted by the meta-atoms, the total radiated field can be expressed as the superposition of radiation from meta-atoms located at positions np:

(4) $$\begin{array}{c} U\left(r\right)=U^{\left(i\right)}\left(r\right)+\sum A_{n}F_{meta}\left(np\right)G_{meta}\left(r,np\right) \end{array}$$

Here:

(5) $$\begin{array}{c} A_{n}=U^{\left(i\right)}\left(np\right)+\sum U^{\left(i\right)}\left(mp\right)F_{meta}\left(np\right)G_{meta}\left(np,\;mp\right)+\cdots \end{array}$$

The first term of $A_{n}$ represents the direct excitation of the *n*-th meta-atom by the incident wave, while the second term represents the first-order excitation of the *n*-th meta-atom by the *m*-th meta-atom, and so forth. Therefore, $A_{n}$ represents the total excitation source of the *n*-th meta-atom.

If all meta-atoms are identical, the product $A_{n}F_{meta}$ is the same for each meta-atom and can be treated as a constant. For oblique incidence, additional spatial phase factors associated with the incident angle must be included. For simplicity, normal incidence is assumed, and Equation (4) can be rewritten as:

(6) $$\begin{array}{c} U\left(r\right)=U^{\left(i\right)}\left(r\right)+A_{n}F_{meta}\sum G_{meta}\left(r,np\right) \end{array}$$

Applying the Floquet’s theorem to the periodic sources [20], the field can be expressed as:

(7) $$\begin{array}{c} \sum G_{meta}\left(r,np\right)=\sum B_{m}e^{j{\vec{k}}_{⊥,\;m}\cdot {\vec{r}}_{⊥}}e^{j{\vec{k}}_{\|,\;m}\cdot {\vec{r}}_{\|}} \end{array}$$

Here:

(8) $$\begin{array}{c} B_{m}=\frac{1}{p}\int G_{meta}\left({\vec{r}}_{\|},{\vec{r}}_{⊥}\right)e^{-j{\vec{k}}_{\|}\cdot {\vec{r}}_{\|}}d{\vec{r}}_{\|} \end{array}$$

(9) $$\begin{array}{c} \left|{\vec{k}}_{\|,\;m}\right|=\frac{2\pi m}{p} \end{array}$$

(10) $$\begin{array}{c} {\vec{k}}_{⊥,\;m}=\pm \sqrt{{\left|\vec{k}\right|}^{2}-{\left|{\vec{k}}_{\|,\;m}\right|}^{2}} \end{array}$$

where $\vec{k}$ is the incident wave vector. The directions parallel and perpendicular to the metamaterial array are denoted by ‖ and ⊥, respectively. $B_{m}$ denotes the amplitude of the *m*-th order Floquet plane wave determined by meta-atom radiation, and *m* is an integer.

We assume the incident wavelength is larger than the lattice period. In the case where only the fundamental resonance is considered and sidelobe radiation is negligent, then only the zeroth-order (m=0) Floquet mode has contribution in the far field, and Equation (6) reduces to:

(11) $$\begin{array}{c} U\left(r\right)=U^{\left(i\right)}\left(r\right)+A_{n}{B_{0}F}_{meta}e^{\pm j\vec{k}\cdot \vec{r}} \end{array}$$

We assume that all meta-atoms are identical single-layer, thin metallic structures. Under this condition, the effective scattering response $F_{meta}$ is identical for all meta-atoms and is symmetric in the two propagation directions. If the incident wave propagation direction is positive (+) (the opposite direction denoted as negative (− )), Equation (11) can be written as:

(12) $$\begin{array}{c} U^{+}=U^{\left(i\right)}+U_{0}^{\left(s\right)}e^{j{\vec{k}}_{+}\cdot {\vec{r}}_{+}} \end{array}$$

(13) $$\begin{array}{c} U^{-}=U_{0}^{\left(s\right)}e^{j{\vec{k}}_{-}\cdot {\vec{r}}_{-}} \end{array}$$

Here, $U_{0}^{\left(s\right)}$ denotes the total radiation strength of each meta-atom after accounting for multiple excitation, which is given by:

(14) $$\begin{array}{c} U_{0}^{\left(s\right)}=A_{n}{B_{0}F}_{meta} \end{array}$$

Recognizing that the positive propagating electromagnetic field is the transmitted wave, while that along the negative direction is the reflected wave, the governing equations describing electromagnetic wave modulation by the metamaterial can be obtained:

(15) $$\begin{array}{c} t=i+s_{+} \end{array}$$

(16) $$\begin{array}{c} r=s_{-} \end{array}$$

where *t*, *r*, and *i* denote the transmitted, reflected, and incident electric fields, respectively, and $s_{+}$ and $s_{-}$ represent the radiation fields emitted by the metamaterial into the opposite directions.

According to Equations (11)–(13), it is evident that resonant radiation must be symmetric in opposite directions, implying opposite momenta and equal energy in the positive and negative direction. Thus, the constraint conditions associated with the modulation can be derived as:

(17) $$\begin{array}{c} \left|s_{-}\right|=\left|s_{+}\right|=s \end{array}$$

(18) $$\begin{array}{c} s_{+}\left(r\right)=s_{-}\left(-r\right) \end{array}$$

Rewrite Equation (15) as:

(19) $$\begin{array}{c} s_{+}=t+\left(-i\right) \end{array}$$

It shows that the electromagnetic radiation emitted by the metamaterial consists of two components: −i, which exactly cancels the incident field, and *t*, which propagates forward as the transmitted wave. This result is fully consistent with the classical extinction theorem, which is here reformulated with metamaterials as the scattering object.

### 3.2. Reconstructed Description of Electromagnetic Wave Modulation by Metamaterials

**Figure 6:**
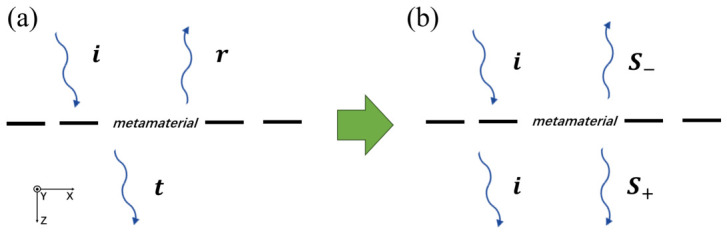
Descriptive diagram of electromagnetic wave modulation by metamaterials. (**a**) Traditional description. (**b**) Reconstructed description.

Based on the modulation relations Equations (15)–(18), this section reconstructs the description of the electromagnetic wave modulation process in metamaterials. As illustrated in Figure 6, we assume a single-layer metamaterial divided the whole space into an incident half-space and a transmission half-space. The half-space toward which the incident wave propagates to metamaterials is defined as the incident half-space (upper half-space in Figure 6), while the opposite region is defined as the transmission half-space. Figure 6a is the conventional description of electromagnetic modulation by metamaterials, and Figure 6b is the reconstructed description from the perspective of the scattering theorem.

In the traditional description, an incident field *i* impinges on the metamaterial, which splits the incident wave into a transmitted wave *t* and a reflected wave *r*. Consequently, the field in the incident half-space consists of the incident wave *i* and the reflected wave *r* propagating in opposite directions, while the field in the transmission half-space contains only the transmitted wave *t*. Within this picture, the amplitudes and phases of the three waves imply a possible design freedom that may allow for independent control through structural engineering. However, as shown above, the incident, reflected, and transmitted waves are mutually constrained, with their amplitudes and phases subject to intrinsic limitations. Therefore, the conventional description requires refinement to incorporate these constraints.

According to the governing equations describing metamaterial modulation (Equations (15) and (16)) and the associated constraint conditions (Equations (17) and (18)), the modulation process can be reformulated as shown in Figure 6b. It can be interpreted as the incident field *i* being distributed throughout the entire space. Under the incident field, the metamaterial radiates, via its induced resonant dipoles, equal-amplitude, equal-phase fields into the two half-spaces in opposite directions. These radiated fields are denoted as $s_{+}$ and $s_{-}$. As a result, the total field in the incident half-space is the superposition of the incident field *i* and the radiated field $s_{-}$, while that in the transmission half-space is the superposition of the incident field *i* and the radiated field $s_{+}$.

Compared with the conventional picture, the reconstructed description retains it as a special case while rendering the intrinsic constraints explicit. The conventional representation follows directly from Equation (16), where the correlation of the radiated fields encodes the mutual relations among *i*, *r*, and *t*.

Furthermore, from the perspective of energy conservation, the difference between the sum of the individual energies of the incident field *i* and the radiated field $s_{+}$ and the energy of their coherently superposed field must be equal to the energy carried by the radiated field $s_{-}$ (or *r*) plus the thermal loss associated with the resonant dipoles. This condition imposes strict constraints on the relative amplitude and phase of the radiated field $s_{+}$ with respect to the incident field *i*. These constraints ultimately manifest as the amplitude–phase relations between the transmitted wave *t* and the reflected wave *r*.

The present description is established for electrically resonant metamaterials. In addition to electric resonances, metamaterials can also support magnetic resonances. For magnetically resonant metamaterials, a possible approach is to decompose the structure into coupled multilayer units and then apply the present description to each individual layer. This strategy provides a potential route toward constructing a corresponding description for magnetically resonant metamaterials.

The present description is established for single-layer metallic resonant metamaterials. In addition to this class, there also exist single-layer dielectric resonant metamaterials. For the latter, the two interfaces of the dielectric layer can be regarded as a coupled bilayer system. Accordingly, such metamaterials can be decomposed into multiple coupled layers through a multilayer coupling expansion, after which the present description may be applied to each individual layer. This approach provides a feasible route toward constructing a corresponding description for dielectric resonant metamaterials.

## 4. Two Corollaries Based on the Reconstructed Description

### 4.1. Coupling Relation Between the Transmitted and Reflected Waves

From the phenomena discussed in Section 2 and the reconstructed description presented in Section 3, it is evident that the transmitted and reflected electric fields satisfy a definite and stringent relationship. In this subsection, this relationship is explicitly derived.

Starting from Equations (15) and (16), one obtains:

(20) $$\begin{array}{c} t\left(\infty \right)=i\left(\infty \right)+s_{+}\left(\infty \right) \end{array}$$

(21) $$\begin{array}{c} r\left(-\infty \right)=s_{-}\left(-\infty \right) \end{array}$$

Further combining Equations (17) and (18) yields:

(22) $$\begin{array}{c} t\left(\infty \right)=i\left(\infty \right)+r\left(-\infty \right) \end{array}$$

By expanding the position variables into spatial phase factors, the expression can be rewritten as:

(23) $$\begin{array}{c} \left|t\right|e^{j∠t}e^{jk\infty }=\left|i\right|e^{jk\infty }+\left|r\right|e^{j∠r}e^{-jk\left(-\infty \right)} \end{array}$$

where ∠t and ∠r denote the phase shifts introduced by the metamaterial, while k∞ and $k\left(-\infty \right)$ represent the phase accumulation associated with spatial propagation. The reference phase is defined such that the incident field *i* has zero phase at the position of the metamaterial. Equation (23) can be rewritten as:

(24) $$\begin{array}{c} \left|t\right|e^{j∠t}=\left|i\right|+\left|r\right|e^{j∠r} \end{array}$$

By normalizing the amplitude of the incident wave, *t* and *r* can be identified as the transmission and reflection coefficients, respectively:

(25) $$\begin{array}{c} t=1+r \end{array}$$

This expression shows that, for a single-layer metamaterial, the transmission coefficient differs from the reflection coefficient only by a unit offset in the real part. In other words, the transmission and reflection coefficients are not independent quantities but are rigidly coupled through this relation. This result demonstrates that, at the field level, transmitted and reflected waves in a single-layer metamaterial obey a strict relation that does not depend on the specific geometry or morphology of the meta-atoms.

In contrast, in conventional approaches such as S-parameter constraints, transmission-line models, and transfer-matrix methods, the transmission and reflection coefficients are typically related indirectly through intermediate quantities (e.g., effective impedance) and are simultaneously constrained by general principles such as energy conservation. However, these approaches do not provide an explicit and direct intrinsic coupling relation between the transmission and reflection coefficients.

### 4.2. Amplitude–Phase Relations of the Reflected and Transmitted Waves

Let the absorption capability of the metamaterial be characterized by the absorption rate *A*. From energy conservation, one obtains:

(26) $$\begin{array}{c} tt^{*}+\;rr^{*}=1-A \end{array}$$

Substituting Equation (25) into Equation (26) yields:

(27) $$\begin{array}{c} r+r^{*}+2\;rr^{*}+A=0 \end{array}$$

By expressing the reflection coefficient as $r=\left|r\right|e^{j∠r}$ and substituting it into Equation (27), the expression can be simplified to:

(28) $$\begin{array}{c} \left|r\right|=\frac{-cos\left(∠r\right)\pm \sqrt{{cos}^{2}\left(∠r\right)-2A}}{2} \end{array}$$

Here, both signs ± correspond to mathematically valid solutions. The physically admissible branch can be determined by the scattering characteristics of the system. Specifically, the negative branch applies when no side lobes are present in the scattering pattern, whereas the positive branch is selected when side lobes appear.

Therefore, the branch selection is not arbitrary but is directly linked to the presence or absence of higher-order scattering features. This provides a clear and practical criterion for identifying the physically relevant solution. The detailed physical mechanism underlying this correspondence will be investigated in future work.

When A=0, only one nonzero solution remains, and the other branch can be unambiguously excluded as a spurious solution. In this case, one obtains:

(29) $$\begin{array}{c} \left|r\right|=-cos\left(∠r\right) \end{array}$$

Following the same procedure, the corresponding relation for the transmitted wave can be derived:

(30) $$\begin{array}{c} \left|t\right|=\frac{cos\left(∠t\right)\pm \sqrt{{cos}^{2}\left(∠t\right)-2A}}{2} \end{array}$$

For the transmission coefficient, the branch selection does not need to be specified independently. Once the physically admissible branch of the reflection coefficient is determined (e.g., based on the presence or absence of side lobes), the corresponding transmission branch is uniquely fixed by the intrinsic transmission–reflection coupling relation derived in this study.

Physically, this reflects the different roles of the two channels: the reflected wave is directly governed by the resonant response of the meta-atoms, leading to a clear branch selection criterion, whereas the transmitted wave results from the coherent superposition of the incident field and the resonant scattering field. As a result, its branch is not independently chosen but is constrained by the overall coupling relation. 

When A=0, this relation reduces to:

(31) $$\begin{array}{c} \left|t\right|=cos\left(∠t\right) \end{array}$$

The above results demonstrate that, in single-layer metamaterials, the reflected and transmitted waves strictly obey Equations (28) and (30). In the absence of absorption, both relations reduce to cosine-type dependencies (Equations (29) and (31)), which are fully consistent with the analysis presented in Section 2. This consistency indicates that, for both transmission and reflection, the phase and amplitude are in one-to-one correspondence and can only take restricted values. Importantly, this correspondence is independent of the specific geometry of the meta-atoms.

In contrast, in conventional approaches such as S-parameter constraints, transmission-line models, and transfer-matrix methods, the amplitude and phase of the transmission and reflection waves are typically determined through intermediate variables or structural parameters. Although phase information can be obtained in transfer-matrix formulations, it mainly reflects inter-layer coupling in multilayer systems and does not provide an explicit amplitude–phase coupling relation for a single-layer metamaterial.

Therefore, these methods do not establish a direct and intrinsic correspondence between amplitude and phase. By comparison, the present results reveal a one-to-one amplitude–phase relation with restricted values, representing a more explicit and geometry-independent constraint at the field level.

### 4.3. Complex-Plane Representation and Potential Implications

**Figure 7:**
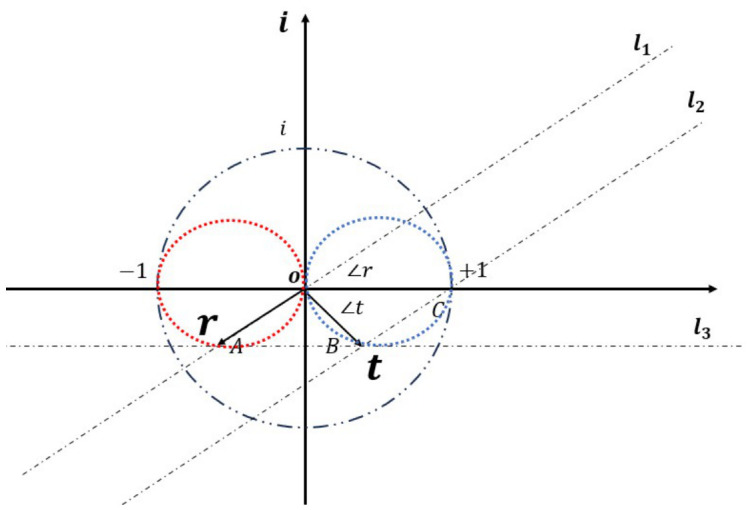
The complex plane distribution diagram of the relationship between the transmission and reflection coefficients.

To clearly illustrate the relationship between the reflected and transmitted waves, this section adopts a complex-plane representation to visualize their intrinsic correlation. For the reflection coefficient, using the phase ∠r and the amplitude relation $\left|r\right|=-cos\left(∠r\right)$, the vector representation of the reflection coefficient *r* in the complex plane is constructed and referred to as the reflection vector. Similarly, based on ∠t and $\left|t\right|=cos\left(∠t\right)$, the vector representation of the transmission coefficient *t* is defined as the transmission vector. The resulting geometric construction is shown in Figure 7.

In Figure 7, $\vec{OA}$ denotes the reflection vector, $\vec{OB}$ denotes the transmission vector, and $\vec{OC}$ is the unit vector along the real axis. The line $l_{3}$ passes through points *A* and *B*, $l_{2}$ passes through points *C* and *B*, and $l_{1}$ passes through points *A* and *O*. The red circle represents the admissible values of the reflection vector, while the blue circle represents the admissible values of the transmission vector.

From Equation (25), one obtains:

(32) $$\begin{array}{c} \vec{OB}=\vec{OA}+\vec{OC} \end{array}$$

According to the parallelogram law of vector addition, the quadrilateral OABC is a parallelogram. Consequently, $\bar{AB}=1$ and ∠r=∠OAB. Considering the relation $\left|r\right|=-cos\left(∠r\right)$, one finds:

(33) $$\begin{array}{c} \bar{OA}=cos\left(∠OAB\right)=\bar{AB}cos\left(∠OAB\right) \end{array}$$

Ecidently, ∠AOB is a right angle, indicating that the reflection and transmission vectors are orthogonal to each other. In other words, the reflection and transmission coefficients differ in phase by 90°. This result is fully consistent with the phenomenon revealed in Section 2.

In summary, the reflection coefficient is confined to a circle in the complex plane whose diameter lies on the negative real axis from 0 to −1 (the red circle in Figure 7), while the transmission coefficient lies on a circle whose diameter spans from 0 to 1 on the real axis (the blue circle in Figure 7). Moreover, the two vectors are strictly orthogonal.

This distribution indicates that, for single-layer metamaterials, the accessible phase range becomes limited when the amplitude approaches high values. Breaking this constraint requires the introduction of multiple resonant couplings, such as interlayer coupling in multilayer metamaterials, where more complex behaviors are expected and remain to be explored.

Based on these intrinsic relations between reflection and transmission, several potential research directions can be envisaged. Specifically, the deterministic coupling between the two channels implies that they are not independently tunable but intrinsically constrained by the same underlying relations. This feature suggests that one channel (e.g., transmission) can be designed to achieve a target functionality, while the other (e.g., reflection), being inherently linked, may serve as a correlated signal for monitoring or feedback.

**Figure 8:**
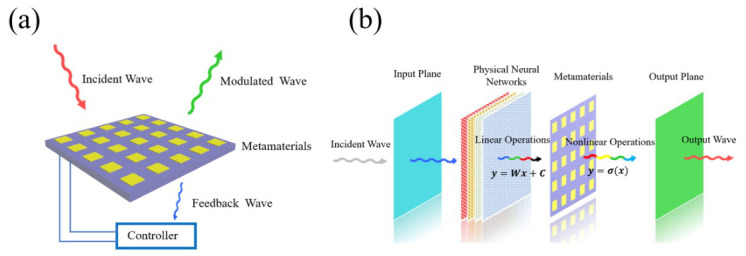
Schematics illustrating the potential applications of the coupling relationships. (**a**) Intelligent metamaterials and information metamaterials. (**b**) Diffractive neural networks.

From this perspective, the metamaterial system can be interpreted as a self-constrained system, where the output channels are intrinsically coupled rather than independently controlled. This property may provide a new perspective for designing information metamaterials and intelligent metamaterials, in which internal correlations between channels could be exploited for feedback or regulation. A schematic illustration is provided in Figure 8a, where an incident wave interacts with the metamaterial; the reflected wave can be utilized as a modulation channel, while the transmitted wave, due to its intrinsic relation with the reflection, may serve as a feedback signal to guide the system response.

Alternatively, since the phase and amplitude are constrained by trigonometric relations, the output fields are not independently variable but follow a coupled mapping determined by these relations. This intrinsic coupling introduces a structured dependence between the input and output, which may effectively act as a built-in nonlinear mapping.

From this viewpoint, such amplitude–phase coupling could be utilized in nonlinear operations. In particular, it may provide a possible mechanism for realizing activation-like responses in physical neural networks (also known as diffractive neural networks), or for nonlinear information encoding in information metamaterials. A schematic diagram is shown in Figure 8b, where the incident wave first passes through an input plane and undergoes linear transformations within a diffractive neural network. Subsequently, a properly designed metamaterial layer, governed by the intrinsic amplitude–phase coupling, may function as an activation-like element to introduce nonlinearity, and the resulting electromagnetic field is then collected at the output plane.

## 5. Case Studies for Verifying the Proposed Description

To verify the newly proposed description of the electromagnetic-wave modulation process in metamaterials, this section examines several consequences derived from the preceding analysis and validates them through specific examples.

The verification strategy is as follows: For a given metamaterial, the transmission–reflection relation expressed in Equation (25) is used to calculate the transmittance from the reflection coefficient, while the amplitude–phase relation in Equation (30) is used to calculate the transmittance from the phase. The transmittance is defined as $T=tt^{*}$.

From Equation (25), one obtains:

(34) $$\begin{array}{c} T=1+r+r^{*}+rr^{*} \end{array}$$

From Equation (30), one obtains:

(35) $$\begin{array}{c} T={\left(\frac{cos\left(∠t\right)\pm \sqrt{{cos}^{2}\left(∠t\right)-2A}}{2}\right)}^{2} \end{array}$$

To avoid ambiguity associated with the elimination of spurious solutions, only the case of A=0 is considered. In this situation, the alternative solution reduces to zero and can be naturally excluded by selecting the nonzero solution:

(36) $$\begin{array}{c} T={\;cos}^{2}\left(∠t\right) \end{array}$$

**Figure 9:**
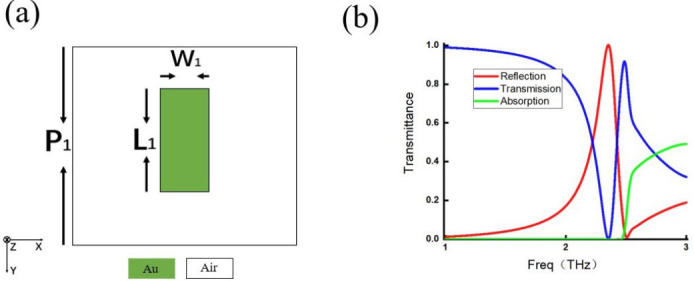
(**a**) Strip-shaped meta-atom with $P_{1}=80\;\mu$m, $L_{1}=50\;\mu$m, and $W_{1}=8\;\mu$m. (**b**) Reflection efficiency curve (red line), transmission efficiency curve (blue line), and absorption efficiency curve (green line) with incidence of $30^{\circ }$.

**Figure 10:**
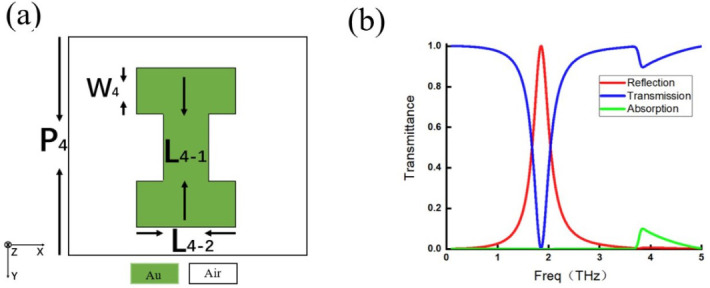
(**a**) I-shaped meta-atom with $P_{4}=80\;\mu$m, $L_{4-1}=40\;\mu$m, $L_{4-2}=40\;\mu$m, and $W_{4}=6.4\;\mu$m. (**b**) Reflection efficiency curve (red line), transmission efficiency curve (blue line), and absorption efficiency curve (green line).

**Figure 11:**
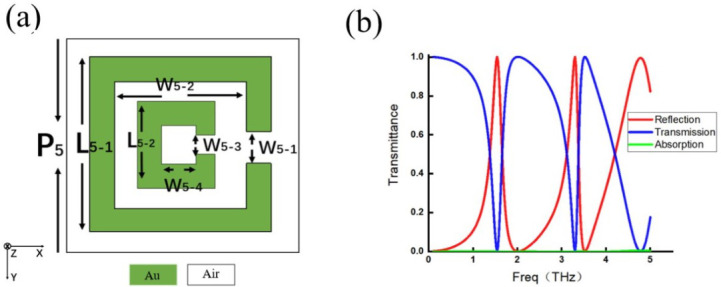
(**a**) Complex split ring meta-atom with $P_{5}=40\;\mu$m, $L_{5-1}=30\;\mu$m, $L_{5-2}=15\;\mu$m, $W_{5-1}=4.8\;\mu$m, $W_{5-2}=20.4\;\mu$m, $W_{5-3}=2.4\;\mu$m, and $W_{5-4}=10.2\;\mu$m. (**b**) Reflection efficiency curve (red line), transmission efficiency curve (blue line), and absorption efficiency curve (green line).

**Figure 12:**
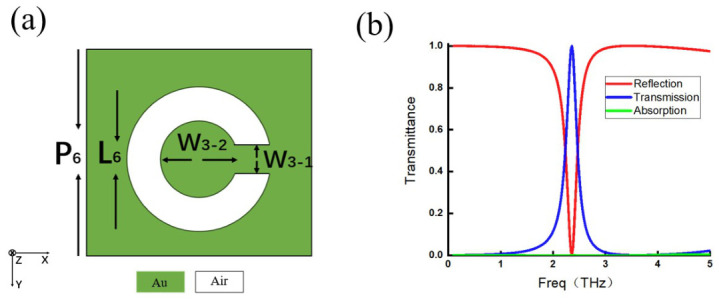
(**a**) Slit split ring meta-atom with $P_{6}=40\;\mu$m, $L_{6}=25\;\mu$m, $W_{5-1}=4\;\mu$m, and $W_{5-2}=1.7\;\mu$m. (**b**) Reflection efficiency curve (red line), transmission efficiency curve (blue line), and absorption efficiency curve (green line).

Four representative single-layer metamaterials are selected for discussion. Each metamaterial features a distinct meta-atom configuration: one is a simple strip-shaped structure (Figure 9a); the second is a more complex I-shaped structure (Figure 10a); the third is a split ring structure with an additional current-loop response (Figure 11a); and the last is a slit-type structure that differs fundamentally from the previous three (Figure 12a). These four configurations can represent metallic inclusion structures and their complementary aperture structures, simple and complex geometries, and straight and split ring structures. For the simple strip-shaped metamaterial, an oblique incidence of $30^{\circ }$ is considered to extend the analysis beyond the limitation of normal incidence.

The transmission spectra of the four structures are shown in Figure 9b, Figure 10b, Figure 11b, and Figure 12b. In these figures, the red curve represents reflectance, the blue curve represents transmittance, and the green curve represents absorptance, which is calculated as 1−R−T and characterizes the energy not distributed into either the reflected or transmitted directions.

**Figure 13:**
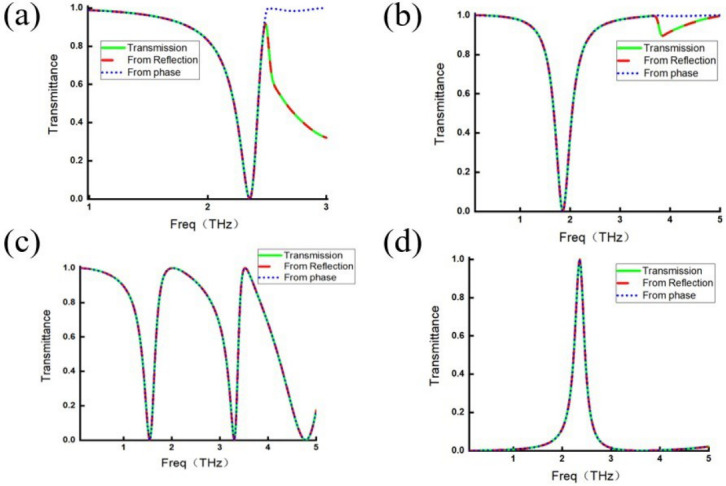
Transmission efficiency curve (green line) calculated from the reflection coefficient (red line) and calculated from the transmission coefficient phase (blue line). (**a**) Strip-shaped metamaterial. (**b**) I-shaped metamaterial. (**c**) Complex split ring metamaterial. (**d**) Slit split ring metamaterial.

For each metamaterial, Figure 13 compares three transmittance curves: the transmittance derived from the reflection coefficient (Equation (34)), that calculated from the phases (Equation (36)), and the directly simulated transmission.

It can be seen that the analytically calculated transmittance curves (blue and red lines) agree well with the simulated transmission spectra (green line) commonly for the four different metamaterials; however, small discrepancies appear in limited frequency intervals, such as the higher-frequency portion beyond 2.5 THz in Figure 13a and beyond 3.5 THz in Figure 13b. In these regions, the discrepancy arises from the transmittance calculated using the phase relation. This deviation originates from the assumption A=0 underlying Equation (36), but the absorptance in these frequency ranges is nonzero actually as demonstrated evidently in Figure 9b and Figure 10b, indicating that part of the energy is neither reflected nor transmitted. This energy may be attributed to scattering or thermal loss. So, for such absorption frequency bands, Equation (35) can be used to correct the result after excluding spurious solutions. However, as discussed earlier, apart from empirical validation, no general criterion for eliminating spurious solutions is provided in the present study. This issue requires further investigation, and the discussion here is therefore limited.

Apart from these cases, the calculated transmittance agrees well with the actual transmittance for all structures. Moreover, the transmittance derived from the reflection coefficient using Equation (34) remains valid even in frequency regions where absorption is nonzero. These observations confirm the validity of the two corollaries derived earlier and, to some extent, support the credibility of the reconstructed description of the electromagnetic-wave modulation process in metamaterials proposed in this study.

## 6. Summary and Conclusions

This study begins with an analysis of the frequency-dependent amplitude and phase of the transmission and reflection coefficients of three representative metamaterials. By employing a complex-plane representation, a general and geometry-independent rule governing the transmission and reflection waves—independent of the specific meta-atom geometry—is revealed.

Based on the scattering theory described by Green’s functions, the mathematical expression of this rule is then derived, and the conventional description of the electromagnetic wave modulation process in metamaterials is reformulated in a more general form. In this context, the manifestation of the extinction theorem in metamaterials is also discussed.

Subsequently, two corollaries are derived from the reformulated description: the coupling relation between the transmission and reflection coefficients, and the amplitude–phase coupling relations of the transmission and reflection waves. The distributions and correlations of these waves are further summarized using a complex-plane representation. Potential applications of these coupling relations are briefly discussed as illustrative implications of the proposed theoretical results, including intelligent metamaterials, information metamaterials, and diffractive neural networks. The revealed coupling relations are of significant importance for the fundamental understanding and rational design of micro/nano devices.

Finally, several specific metamaterial structures are examined, and their transmittance calculated from the derived relations is compared with the actual results, demonstrating the validity of both the corollaries and the reformulated description. This study provides a general theoretical framework for understanding electromagnetic wave modulation in metamaterials and establishes intrinsic constraints and coupling relations that may serve as a foundation for further investigations of multilayer coupling and multi-resonant coupling in metamaterial systems.

## Data Availability

The data that support the findings of this study are available from the corresponding author upon reasonable request.
